# Mesopore Controls the Responses of Blood Clot‐Immune Complex via Modulating Fibrin Network

**DOI:** 10.1002/advs.202103608

**Published:** 2021-11-24

**Authors:** Shiyu Wu, Zhengjie Shan, Lv Xie, Mengxi Su, Peisheng Zeng, Peina Huang, Lingchan Zeng, Xinyue Sheng, Zhipeng Li, Gucheng Zeng, Zhuofan Chen, Zetao Chen

**Affiliations:** ^1^ Hospital of Stomatology Guanghua School of Stomatology Sun Yat‐sen University and Guangdong Provincial Key Laboratory of Stomatology Guangzhou 510055 China; ^2^ Department of Microbiology Zhongshan School of Medicine Sun Yat‐sen University Guangzhou 510080 China; ^3^ Clinical Research Center Department of Medical Records Management Guanghua School of Stomatology Hospital of Stomatology Sun Yat‐sen University Guangzhou 510055 China

**Keywords:** clot‐immune, fibrin network, fibrinogen, mesopore

## Abstract

Formation of blood clots, particularly the fibrin network and fibrin network‐mediated early inflammatory responses, plays a critical role in determining the eventual tissue repair or regeneration following an injury. Owing to the potential role of fibrin network in mediating clot‐immune responses, it is of great importance to determine whether clot‐immune responses can be regulated via modulating the parameters of fibrin network. Since the diameter of D‐terminal of a fibrinogen molecule is 9 nm, four different pore sizes (2, 8, 14, and 20 nm) are rationally selected to design mesoporous silica to control the fibrinogen adsorption and modulate the subsequent fibrin formation process. The fiber becomes thinner and the contact area with macrophages decreases when the pore diameters of mesoporous silica are greater than 9 nm. Importantly, these thinner fibers grown in pores with diameters larger than 9 nm inhibit the M1‐polorazation of macrophages and reduce the productions of pro‐inflammatory cytokines and chemokines by macrophages. These thinner fibers reduce inflammation of macrophages through a potential signaling pathway of cell adhesion‐cytoskeleton assembly‐inflammatory responses. Thus, the successful regulation of the clot‐immune responses via tuning of the mesoporous pore sizes indicates the feasibility of developing advanced clot‐immune regulatory materials.

## Graphical abstract

Tuning mesoporous silica pore size is found to affect blood clot coagulation and change fibrin network structure through regulating fibrinogen adsorption. The regulated fibrin network further modulates the immune response of macrophage via a potential pathway of cell adhesion‐cytoskeleton assembly‐inflammatory response, indicating a feasible strategy to develop advanced clot‐immune regulatory materials.

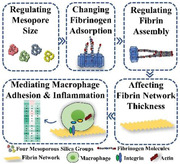

## 1 Introduction

Blood clotting and initiation of inflammation are the most typical early events for tissue repair or regeneration. Blood clotting begins with a vascular spasm and platelet aggregation, followed by a clotting cascade and fibrinogen activation, leading to the formation of a fibrin network.^[^ ^1^ ^]^ This fibrin network not only serves as a transient extracellular matrix for cell adhesion, proliferation, and differentiation, but is also involved in modulating inflammation and innate immunity.^[^ ^2^, ^3^ ^]^ A sustained inflammatory response may result in impairment of tissue repair, but a timely transition to the reparative phase will promote tissue regeneration.^[^ ^4^ ^]^ Thus, inflammatory responses are required to be well orchestrated to avoid over‐reactive inflammation for a successful tissue repair, and the modulation of early blood clot formation and blood clot formation‐associated inflammatory response is of critical importance for a successful reparative or regenerative process.

However, it remains challenge for a well‐modulated regulation for the interaction between clotting and inflammation,^[^ ^5^, ^6^ ^]^ and this clot‐immune complex. It has been shown that changing the parameters (e.g., diameter) of the fiber may promote inflammatory responses.^[^ ^7^, ^8^ ^]^ Particularly, inappropriate fiber thickness during blood clotting may jeopardize long‐term regenerative outcomes.^[^ ^9^, ^10^, ^11^ ^]^ When the original fiber thickness is increased, the efficiency of bone regeneration becomes considerably decreased.^[^ ^10^, ^11^ ^]^ Importantly, decreased fiber diameter would favor a thread‐like or net‐like morphology of fibrin, which further increased expression of M2 makers of macrophages (MΦ) and improved the bone regeneration efficiency.^[^ ^9^ ^]^ Modulating the fiber parameters may serve as an effective strategy for orchestrating clot‐immune complex involving in blood clotting structure and inflammatory responses.

There are three phases in a typical fibrin formation process: 1) nucleus formation, which refers to the formation of dimers via the *α*A interaction of two fibrinogen molecules; 2) linear growth, which refers to the formation of a double‐stranded, half‐staggered protofibril; and 3) lateral growth, which refers to the lateral aggregation of protofibrils that determines the fiber thickness.^[^ ^12^, ^13^ ^]^ Drawing upon this process, materials have been developed to regulate fibrin formation by modulating the duration of the linear growth and lateral aggregation phases.^[^ ^14^ ^]^

First, regulating the concentration (fibrinogen, thrombin) or ratio (fibrinogen: thrombin) of raw materials will form more initial nuclei, which will extend the linear growth phase and reduce the lateral aggregation phase, thus producing thinner fibers.^[^ ^15^, ^16^ ^]^ Second, modifying the nucleation environment (pH and ionic concentration) will interfere with the lateral aggregation process, thus altering the fiber thickness.^[^ ^12^ ^]^ However, for ongoing blood clotting in vivo, it is difficult to regulate the concentration/ratio of raw materials as well as the nucleation environment. Therefore, we propose a strategy to regulate fibrin formation by introducing exogenous nucleus into the system to regulate the linear growth and lateral aggregation.

The fibrinogen molecules, with a length of 47.5 nm and diameter of 6 nm × 9 nm, consist of a central E domain and two outer D domains connecting to the center. To form an exogenous nucleus, the materials need to gather sufficient dimers via the high adsorption ability of fibrinogen. Mesoporous materials are an ideal candidate because of their adjustable mesopore sizes ranging from 2 to 50 nm, which meet the requirement of size threshold of fibrinogen. Therefore, it is of great interest to tune the mesoporous size to regulate the fiber thickness and the responses of clot‐immune complex.

To improve linear growth of fibrin, the terminal D domain is selected as the adsorption target of mesoporous material, and two mesopore sizes below 9 nm (2 and 8 nm, respectively), and two mesopore sizes above 9 nm (14 and 20 nm, respectively) are selected for analysis of differential adsorption. It is assumed that the 14 and 20 nm mesopores may mediate thinner fibers by providing additional nuclei by adsorbing the D domain of fibrinogen, whereas the 2 and 8 nm mesopores may confer little effect on modifying the fiber thickness because of the lack of accommodation of the D domain area.

**Figure 1:**
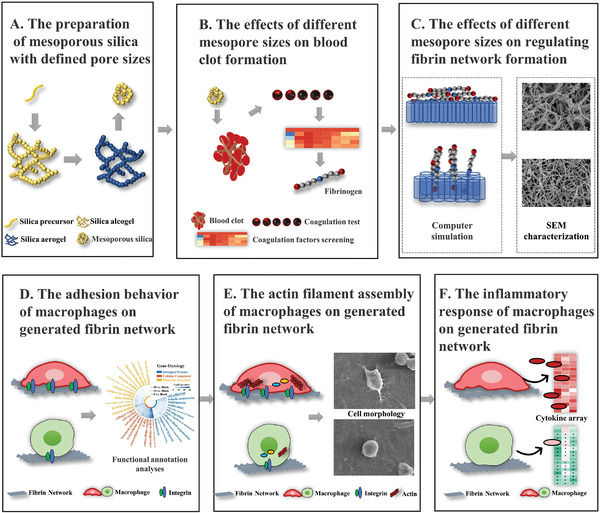
Experimental flow of the study. A) Mesoporous silica with defined pore sizes is prepared using modified sol–gel method. B) Mesopore‐induced clotting time is confirmed in vitro and in vivo, followed by the detection of the key coagulation factor: fibrinogen. C) The effects of mesopore on regulating fibrin network formation and the characteristics of generated fibrin network are investigated using computer simulation, scanning electron microscope (SEM), etc. D) A potential pathway of cell adhesion‐cytoskeleton assembly‐inflammatory response is found using bioinformatic analysis. The adhesion behavior of macrophages on fibrin network is investigated using finite element analysis, RNA‐sequencing (RNA‐seq), real‐time quantitative polymerase chain reaction (RT‐qPCR), and immunofluorescence. E) The cytoskeleton assembly is then evaluated using RNA‐seq, RT‐qPCR, SEM, and immunofluorescence. F) Inflammatory response of macrophages is eventually evaluated using RNA‐seq, RT‐qPCR, cytokine array, enzyme‐linked immunosorbent assay (ELISA), and immunofluorescence.

The effects of fibrin networks with different fiber thickness on the inflammatory response of macrophages are then examined, and the underlying mechanisms by which fibrin with different thickness regulate the inflammatory response of macrophage are unveiled (**Figure** **1**). This study considers blood clots and the early immune response as a single interactive complex. It proposes an effective strategy to manipulate this clot‐immune complex by modulating the parameters of the clot fibrin network, which may pave a new way for the development of advanced clot‐immune regulatory biomaterials.

## 2 Results and Discussion

### 2.1 Preparing Mesoporous Silica Particles with Controlled Mesopore Sizes

**Figure 2:**
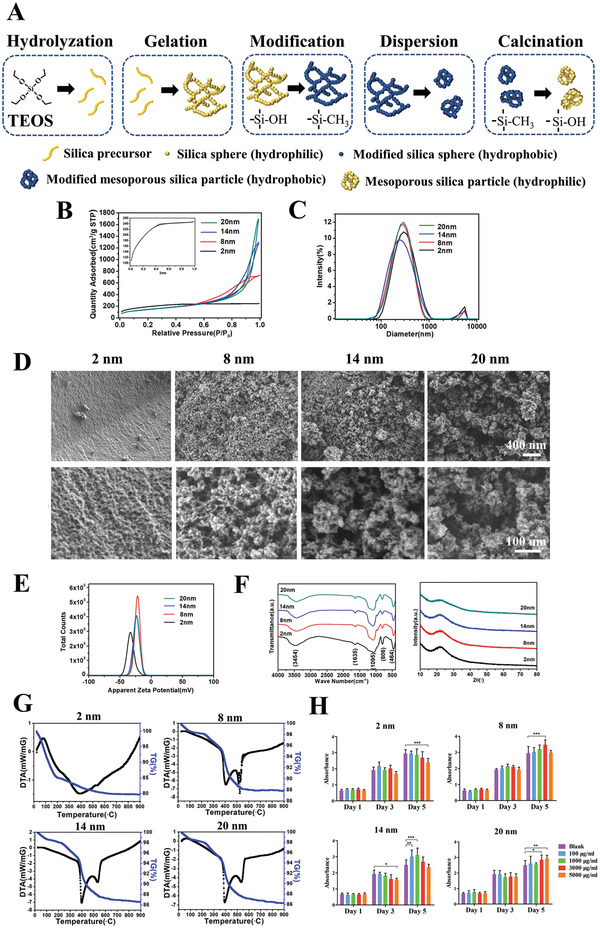
Preparation, morphology, and structure characterization, composition, and cytotoxicity of mesoporous silica particles with controlled mesopore sizes. A) Schematic illustration of the overall preparation procedure for mesoporous silica particles with controlled mesoporous sizes. B) The nitrogen adsorption–desorption isotherm of the prepared mesoporous silica detected by BET. C) Particle size distribution curve of the prepared mesoporous silica detected by Nano Sizer and Zeta‐potential Tester. D) Morphology and structure characterization of the prepared mesoporous silica detected by SEM. E) Zeta potential distribution curve of the prepared mesoporous silica. F) FTIR and XRD analysis of the prepared mesoporous silica. G) TG‐DSC of the prepared mesoporous silica. H) Cytotoxicity test of the prepared mesoporous silica using CCK‐8. Data are presented as means ± s.d.; *n* = 5; *p < 0.05, **p < 0.01, ***p < 0.001 by two‐way ANOVA with Tukey's post hoc test.

The commonly used template method often produces mesoporous materials with a pore size of less than 6 nm.^[^ ^17^, ^18^, ^19^ ^]^ To overcome the limit of the pore size of the template method, a modified sol–gel method is utilized in this study (**Figure** **2**A). In this method, the condensation reaction rate is the main factor affecting the mesopore size, while pH is the key to regulate the condensation reaction rate. The pH affects the polymerization of silicon‐oxygen bonds by influencing the electrification of the silicon source. When pH is greater than the isoelectric point, the polymerization rate increases with pH. The density of the silica gel increased with the increase of polymerization, following the decrease of the porosity. Therefore, a large mesopore size can be generated via the sol–gel method and pH optimization.

In this experiment, the modified sol–gel method is used to prepare silica hydrogels and dried to obtain mesoporous silica aerogels. However, silica hydrogel may produce mesoporous materials with larger particle size and higher collapse risk of the pore structure during drying. Ultrasonic dispersion is included to create a more uniform particle size and thus address these issues. Hexamethyl diethylamine is utilized for methyl hydrophobic modification to reduce the water content of the gel and prevent structural collapse because of the surface tension of water during drying.

With this approach, modified aerogel mesoporous silica nanoparticles with controllable average mesopore sizes of ≈2.43, 8.05, 13.75, and 20.33 nm are successfully developed. This is shown by Brunauer–Emmett–Teller (BET) analysis results (Figure 2B, **Table** **1**), which are close to the required sizes. The average particle size of each group is ≈350–400 nm, which shows that ultrasonic dispersion can yield a lower average particle size (Figure 2C). Scanning electron microscopy (SEM) imaging shows that mesoporous structure is well preserved with no obvious collapse after modification by hexamethyl diethylamine (Figure 2D). Due to the difference in synthesis method, physical and weak phase interactions exist among secondary Si particles in this sol–gel technique produced mesoporous silica, which is prone to be damaged by high voltage electronic beam. ^[^ ^20^, ^21^ ^]^ Therefore, we optimize the shooting method of SEM by keeping a low voltage and using integration mode (Figure S1, Supporting Information). The transmission electron microscope (TEM) detection is also attempted. However, we cannot identify the clear mesopore structure in TEM images, which have also been well documented in other studies (Figure S1, Supporting Information).^[^ ^22^, ^23^ ^]^

**Table 1:** The nitrogen adsorption and desorption test results of mesoporous silica

Sample name	Average pore size [nm]	Pore volume [cm^3^ g^−1^]	Surface area [m^2^ g^−1^]
2 nm	2.43	0.38	667.70
8 nm	8.05	1.12	511.52
14 nm	13.75	1.58	436.33
20 nm	20.33	2.63	510.73

The surface charge of materials might affect protein adsorption, so the zeta potential is also tested. The results show that the materials in each group are negatively charged and have similar charges (Figure 2E). These results suggest that mesoporous silica particles with controlled mesopore size are successfully prepared, and most of physical and chemical parameters of these mesoporous silica particles are generally consistent, which can be applied in the following functional and mechanism studies. To make the manuscript more concise and the terminology more consistent, we name the prepared materials as mesoporous silica.

With regard to the composition, the vibration peaks of Si—O—Si and —OH are detected by Fourier‐transform infrared spectroscopy (FTIR), and the X‐ray diffraction (XRD) results suggest that the material was amorphous (Figure 2F). Thus, the prepared material is an amorphous mesoporous silica material. In mesoporous silica preparation, hexamethyl diethylamine is utilized for methylation modification, which can be toxic to cells and should be removed. Thermogravimetric analysis‐differential scanning calorimetry (TG‐DSC) tests show that the methyl decomposition temperature is ≈535 °C (Figure 2G). The demethylation temperature of the mesoporous nanoparticles is set as 600 °C. FTIR reveals that the asymmetric vibration and bending vibration peaks of 2966 and 850 cm^–1^ of the methyl group disappear after the heat treatment, which proves the complete decomposition of the methyl group (Figure 2F, Figure S2, Supporting Information).

The toxicity results show that a certain amount of toxicity is present at high concentrations (5000 µg mL^−1^), which may be caused by the mesoporous silica (Figure 2H). These results indicate that mesoporous silica with controlled mesopore sizes are successfully prepared and can be applied in the subsequent studies.

### 2.2 The Effects of Mesopore Size on Blood Clot Formation

**Figure 3:**
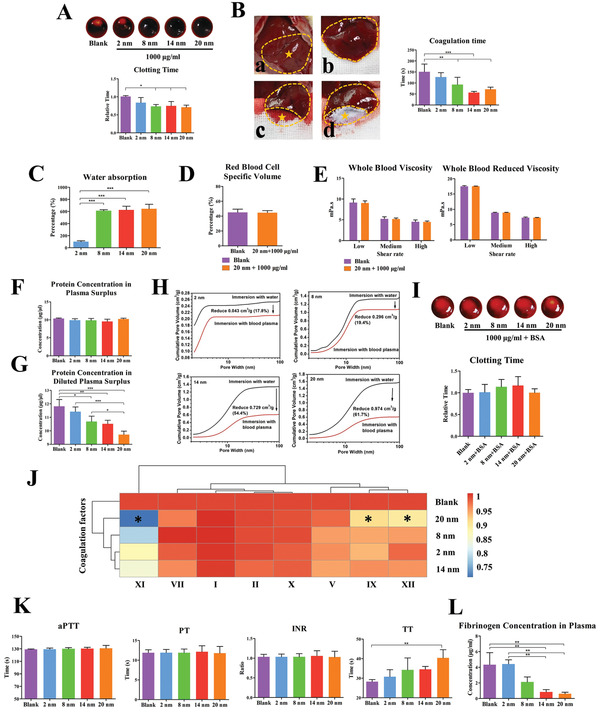
The effects of mesopore size on blood clot formation. A) Coagulation time test in vitro. B) Coagulation time test in vivo using rat liver injury model. a) Exposure of the liver. b) Exposure of the left inner lobe of liver. c) Liver injury in control group. d) Liver injury site covered with mesoporous silica. C) Water absorption capacity test. D) Red blood cell specific volume test. E) Blood viscosity test. F) Protein concentration of plasma surplus after mesoporous silica adsorbed. G) Protein concentration of diluted plasma surplus after mesoporous silica adsorbed. H) Pore volume changes after mesoporous silica adsorbing protein. I) Coagulation time test in vitro using BSA pre‐adsorbed mesoporous silica. J) Plasma coagulation factors concentration after mesoporous silica adsorbing. K) The aPTT, PT, INR, and TT tests using mesoporous silica‐adsorbed plasma. L) Plasma fibrinogen concentration after mesoporous silica adsorbing using fibrinogen ELISA. Data are presented as means ± s.d.; *n* = 3; *p < 0.05, **p < 0.01, ***p < 0.001 by one‐way ANOVA with Tukey's post hoc test or unpaired two‐tailed Student's *t*‐test.

As the initial stage of wound healing, blood clotting quality plays an important role in the long‐term regeneration effect.^[^ ^5^, ^6^ ^]^ Therefore, in the process of clot‐immune regulation, we first clarify the regulatory role of materials on the basic clot formation behavior. To unveil the effect of mesoporous silica on coagulation time, in vitro clotting time test and in vivo liver injury are performed.^[^ ^24^ ^]^ The clotting time of the 8 nm, 14 nm, and 20 nm groups are significantly reduced compared with that of the blank group (**Figure** **3**A,B). These results indicate that the mesopore size of 8–20 nm could accelerate coagulation; this is consistent with a previous study which shows that pore sizes ranging from 5 to 15 nm can shorten the blood clotting time.^[^ ^24^ ^]^

We further explore the mechanism of mesopore‐mediated clot formation. Mesopores may accelerate the coagulation process through water and protein adsorption.^[^ ^24^, ^25^, ^26^ ^]^ We first examine the water absorption capacity of mesopores and find that all mesoporous materials with mesopore sizes of 8, 14, and 20 nm exhibit excellent water absorption capacity (Figure 3C). No significant difference is found between the material groups and blank group in terms of red blood cell specific volume, whole blood viscosity, and reducing viscosity (Figure 3D,E). These results indicate that although the mesoporous material has good water absorption performance, it may not play a central role in reducing the coagulation time.

Since the protein adsorption ability is one of the key mechanisms for mediating blood clotting of mesoporous materials, we test whether the mesopore size affect the protein adsorption capability. We find that the amount of mesoporous silica adsorbed do not change the total concentration of plasma protein (Figure 3F), whereas the amount of protein adsorbed by the material itself increases with the increase of pore size, which is confirmed in the BCA detection of diluted plasma supernatant (Figure 3G). The pore volume is reduced to a 17.8%, 19.4%, 54.5%, and 61.7% in the 2, 8, 14, and 20 nm groups, respectively (Figure 3H). Furthermore, the pro‐coagulant ability of mesoporous silica is abolished when the material are pre‐treated with bovine serum albumin (BSA) (Figure 3I), which suggests that protein adsorption is a key mechanism for coagulation ability mediated by mesoporous silica.

We then analyze several key pro‐coagulation factors using activated partial thromboplastin time (aPTT), prothrombin time (PT), international normalized ratio (INR), and thrombin time (TT) tests, to detect abnormal concentrations of blood coagulation factors. The APTT, PT, and INR tests measure the clot formation rate of intrinsic and extrinsic coagulation pathways. Although the particles adsorb some coagulation factors (Figure 3J), no significant difference is found among aPTT, PT, and INR (Figure 3K); hence, these are not the key coagulation factors that affect mesopore‐mediated clot formation. The thrombin time test is used to measure the time of common pathway of coagulation from fibrinogen to fibrin caused by thrombin. The thrombin time increases as the pore size increases, with the thrombin time of the 20 nm group significantly longer than that of the control group (Figure 3K), suggesting the decreased concentration of fibrinogen adsorbing by mesopores. As the aPTT and PT remained unchanged, whereas the thrombin time increased as the mesopore size increased, mesopore‐adsorbed fibrinogen might serve as a key rate‐limiting step during fibrin formation. This is further confirmed by the enzyme‐linked immunosorbent assay (ELISA) results showing the mesopore‐size‐dependent adsorption of fibrinogen (Figure 3L).

These results indicate that larger mesopore sizes (14 and 20 nm) can accelerate the clot formation rate by adsorbing fibrinogen. The adsorption behavior reveals a mesopore‐size‐dependent effect, which confirms our hypothesis that mesopores of 14–20 nm can adsorb fibrinogen, whereas mesopores of 2 and 8 nm have little effect.

### 2.3 The Effects of Mesopore Size on Regulating Fibrinogen Behaviors

**Figure 4:**
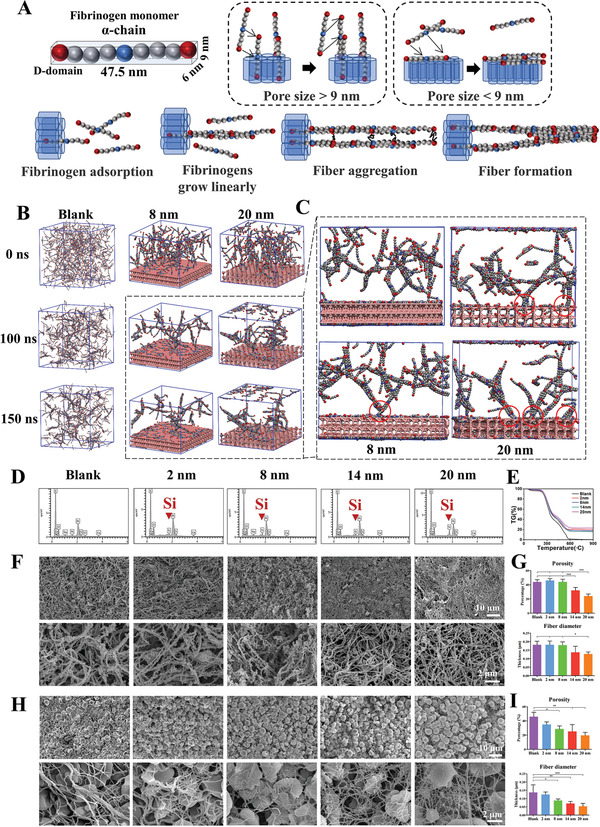
The mesopore sizes above 9 nm generate thinner fibers via regulating fibrinogen behaviors. A) The model fibrinogen monomer *α*‐chain is presented, showing the size of fibrinogen D domain is 6 nm × 9 nm. When the mesoporous size is smaller than 9 nm, the fibrinogen D domain cannot enter the mesopore and the entire fibrinogen structure is adsorbed on the mesoporous structure surface. Opposite situation can be observed in the > 9 nm group. Part of the fibrinogen enters the pore structure, with the remaining part becoming exogenous nucleus. B) Molecular dynamics simulation diagrams of fibrinogen aggregation in the blank, 8 nm, and 20 nm groups. C) The adsorption sites of the 8 and 20 nm groups in enlarged view. The interaction sites of mesoporous silica and fibrin are labeled as red circles. D) Element distribution of mesoporous silica‐regulated fibrin network using SEM‐EDS. E) TG detection of mesoporous silica‐regulated fibrin gel. F) SEM observation of mesoporous silica‐regulated fibrin gel. G) The porosity and diameter measurement of mesoporous silica‐regulated fibrin gel. H) SEM observation of mesoporous silica‐regulated blood clot fibrin network. I) The porosity and diameter measurement of mesoporous silica‐regulated blood clot fibrin network. Data are presented as means ± s.d.; *n* = 3; *p < 0.05, **p < 0.01, ***p < 0.001 by one‐way ANOVA with Tukey's post hoc test.

As mentioned above, the physical size of fibrinogen is 6 nm × 9 nm × 47.5 nm plays critical role in mediating the adsorption ability of fibrinogen and subsequent network formation. When the pore size of the mesoporous material is less than 9 nm, the mesopore of mesoporous materials displays poor fibrinogen adsorption ability, both D domains of fibrinogen are adsorbed only on the very surface, but not inside the pore of the material. As a result, the fibrinogen monomers cannot continue to grow linearly through the “occupied” D domains.^[^ ^24^, ^27^, ^28^ ^]^ Therefore, we hypothesized that a small mesopore size did not have significant effects on changing the fibrinogen behavior and the subsequent fiber network formation (**Figure** **4**A).

This hypothesis is further confirmed in a reactive coarse‐grained molecular dynamics study. Fibrinogens in the 8 nm group are adsorbed on the surface of the material, and the fibrinogen monomer is unable to continue to grow linearly through the adsorbed fibrinogens (Figure 4B,C). Fibrinogens aggregate mainly at the top of the material, and few fibrins falling on the surface of the material is observed. However, the molecular dynamics simulation of the 8 nm group shows that at 100 ns, three fibrinogen molecules fell on the surface of the mesoporous material; at 150 ns, only one fibrinogen molecule remains. This connection differs from the normal growth process of fibrinogen monomer because it floats again during the subsequent growth process. Therefore, the 8 nm mesoporous group does not significantly change the fibrin formation.

In contrast, when the mesopore size is greater than 9 nm, one D domain of fibrinogen can be adsorbed into the mesopore and the other D domain is exposed outside the surface (Figure 4B,C). Importantly, such outside exposed D domain can be regarded as exogenous nucleus, and the free fibrinogen monomers will grow linearly on this exogenous nucleus. The chain‐like fibers may aggregate and thicken through the interaction force in the *α*C domain interactions after linear growth, and it is more difficult for the fiber chains to aggregate, resulting in generations of a thinner fiber, since one end of the chain fibers is stuck in the mesopores. (Figure 4B,C). This theory is well supported by the molecular dynamics simulation (Figure S3A, Supporting Information), which shows that almost all fibrins grow from the mesopore surface and the initially adsorbed fibrinogen is encapsulated by the newly generated fibrin. Therefore, a mesopore size greater than 9 nm can significantly change the fibrin formation to facilitate the production of thinner fibers.

To further verify the change in the mesoporous‐size‐mediated fiber thickness, mesoporous silica is first mixed with plasma and coagulation factors. Energy dispersive X‐ray spectroscopy (EDS) and TG show that the generated fibrin network contain ≈20% silicon (Figure 4D,E), indicating the presence of mesoporous silica inside the fibrin network. Mesopores larger than 9 nm generates denser fibrin networks with smaller porosity and fiber thickness (Figure 4F,G), and higher strength (Figure S3B, Supporting Information). We further test the effects of mesopores on the entire blood clot fibrin network, and similar results are observed as well (Figure 4H,I). The porosity and fiber diameter decrease gradually with the increase of mesopore size (Figure 4H,I), but the clot strength increases with the increase of pore size (Figure S3C,D, Supporting Information).

It is worth to note that the silica particles are not observed in the fibrin network during SEM observation (Figure 4F,H). The possible reason underlying this absence of silica particles is that the material is inside but not on the surface of the fibrin network and cannot be reached via SEM images. Given that TG analysis shows that fibrin network contains ≈20% silicon, it is reasonable to speculate that the fibrin network wraps the particles and contains appreciable amounts of silica particles. This indicates that the direct silica particle effect on following cell response detection may not be significant.

In addition to the direct effect, the silicon ions released by mesoporous silica may also affect the biological function of macrophages. To exclude this effect, we investigate the effect of released silicon ions. After mixing the material and blood in proportion, its extract is collected for silicon ion detection using inductively coupled plasma atomic emission spectrometer (ICP‐AES). No significant difference is found between the concentration of released silicon ion among four material groups (*p* = 0.3981), suggesting the effects of silicon ion may not be in a major role in regulating further cell response (Table S1, Supporting Information).

In summary, the mesoporous silica changes the fibrin formation by adsorbing fibrinogen. The pore size of mesoporous structures is a key parameter of mesoporous silica that changes fibrin formation. Only when the diameter of the mesopore is larger than the D‐end of fibrinogen (> 9 nm) can fibrinogen be adsorbed to the surface upright, and free fibrinogen can aggregate to form chain fibers. Furthermore, because the chain‐like fibers are stuck in the mesopores, lateral aggregation is limited, which leads to the formation of thinner fibers. These results indicate the feasibility of adjusting fibrin formation by changing the mesopore size. It should be noted that the depth of the mesopore may also elicit significant effects. When the pore size is large enough (> 47.5 nm), the fibrinogen may completely enter the pore structure and the D domain could be hidden, resulting in the inhibition on normal coagulation process.

### 2.4 The Effects of Generated Fibrin Network on the Inflammatory Response of Macrophages

While T cells, neutrophils, B cells, may all play important role in mediating the inflammation in immune microenvironment during blood clotting, we and other groups have previously shown that macrophage (MΦ) is one of immune cells with strong regulation ability and excellent plasticity interacting with biomaterials to mediate blood clot formation.^[^ ^29^, ^30^, ^31^ ^]^ Indeed, the timely transition from the macrophage‐mediated M1 phase to the M2 phase promotes tissue repair, whereas a persistent M1 phase may result in fibrous formation.^[^ ^32^ ^]^ Therefore, we focus on macrophage as a model cell to investigate how the generated fibrin network regulates their inflammatory responses.

**Figure 5:**
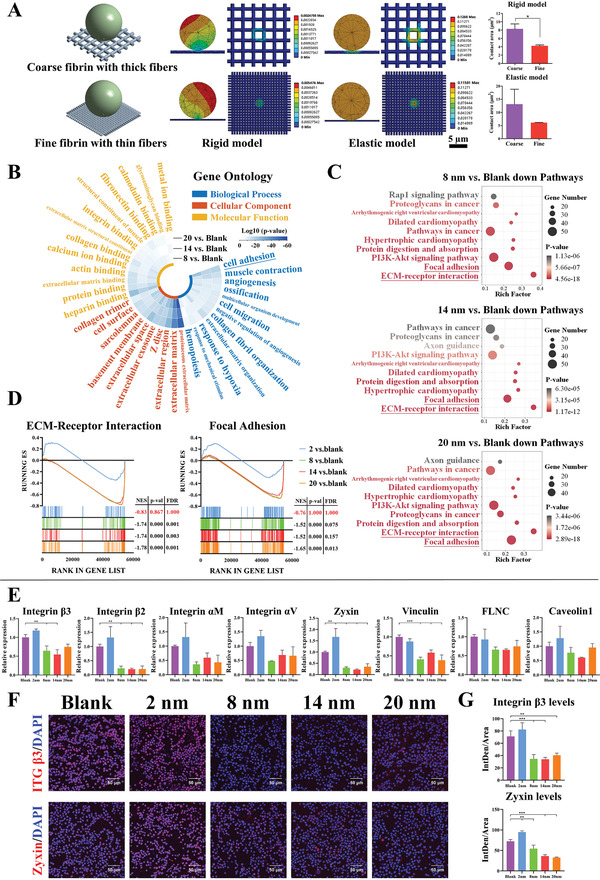
The adhesion behavior of macrophages is inhibited on generated fibrin network with thinner fibers. A) Finite element analysis shows the cell–fibrin contact area in rigid and elastic models. B) GO analysis reveals the Top10 terms enriched in differentially downregulated genes (including 8, 14, and 20 nm vs Blank) for biological process, cellular component, and molecular function. Tile color, log10 (*p*‐value). C) KEGG analysis reveals the Top10 pathways enriched in differentially downregulated genes (including 8, 14, and 20 nm vs Blank). X‐axis, rich factor; Y‐axis, enriched terms; dot sizes, gene number; dot color, *p*‐value. D) GSEA reveals the global difference in 2, 8, 14, and 20 nm vs Blank of ECM‐Receptor Interaction and Focal Adhesion. |NES| > 1, NOM *p*‐val < 0.05 and FDR *q*‐val < 0.25 are considered significant. E) RT‐qPCR results of adhesive receptors and focal adhesion related gene expressions in macrophages cultured on fibrin network. F–G) Representative immunofluorescence images and semi‐quantitative statistical analysis of integrin *β*3 (one of adhesive receptors for fibrin) and zyxin (one of focal adhesion genes) in macrophages cultured on fibrin network. Data are presented as means ± s.d.; *n* = 3; *p < 0.05, **p < 0.01, ***p < 0.001 by unpaired two‐tailed Student's *t*‐test or one‐way ANOVA with Tukey's post hoc test.

Since the adhesion sequences and the interactions with cell adhesive receptors are critically important for fibrinogen to regulate the inflammatory response,^[^ ^33^ ^]^ we postulate that the fibrin network regulates the inflammatory response of macrophages by affecting cell adhesion first. The models based on computer simulation show that the mesopore size regulates fibrin behavior, resulting in the changes in fiber thickness (Figure 4B,C). Finite element analysis show that the fiber diameter decreases with the decrease of the contact area of cell–fibrin (**Figure** **5**A). A model in which cells contact the fibrin network only under gravity shows that the contact area with a coarse fibrin network and a fine fibrin network is 8.3 µm^2^ and 4.2 µm^2^, respectively (Figure 5A). Considering that cells can be pressed onto the fibrin network in vivo, another displacement model, in which cells are squeezed onto the fibrin network by 0.1 µm, shows contact areas of 13.1 and 6.1 µm^2^, respectively (Figure 5A). According to these results, it is reasonable to hypothesize that the fiber thickness decreases with the reduction of the adhesion behavior between macrophages and fibrin network.

To test this hypothesis, we first use RNA‐sequencing (RNA‐seq) to analyze the changes in gene expression. We find that the 8 nm, 14 nm, and 20 nm groups are significantly different from the blank group in terms of their ability to regulate the gene expression of macrophages, with good intra‐group repeatability as shown in principle component analysis (Figure S4A, Supporting Information). However, 2 nm group shows no significant difference with the blank group (Figure S4B,C, Supporting Information); hence, 2 nm group is excluded from the subsequent in‐depth analysis. The differentially downregulated genes of macrophages in the 8, 14, and 20 nm groups, are subjected to the analyses of functional annotation via Gene Ontology (GO) and pathway annotation via Kyoto Encyclopedia of Genes and Genomes (KEGG). The analyses of the top 10 downregulated GO terms show that cell adhesion in each group is the most significant events in the biological processes, which suggests that cell adhesion can be a major downregulated event upon contact with fibrin (Figure 5B). KEGG and Gene Set Enrichment Analysis (GSEA) further show that the first two pathways among the top 10 downregulated pathways in each group are extracellular matrix (ECM)‐receptor interaction and focal adhesion, which are related to cell‐extracellular matrix adhesion and intracellular adhesion structure, respectively (Figure 5C,D, Figure S5A, Supporting Information). To clarify how cell adhesion is limited in the 8, 14, and 20 nm groups, key genes from the leading‐edge subset of above two adhesion pathways are screened out. It is shown that 8, 14, and 20 nm groups display similar profiles of downregulated adhesion genes, but the 14 nm group shows the most significant downregulation of adhesion genes (Figure S5B,C, Supporting Information).

To further verify the downregulation of cell adhesion, main adhesive receptors for fibrin (i.e., integrin *β*3, *β*2, *α*M, and *α*V) and main focal adhesion‐related genes (zyxin, vinculin, filamin‐C, and Caveolin1) are detected by real‐time quantitative polymerase chain reaction (RT‐qPCR) or immunofluorescence. The results show that most of the detected adhesion genes and proteins are downregulated in all three groups (Figure 5E–G). Taken together, these results indicate that the adhesion behaviors of macrophages on the fibrin network generated by 8 nm, 14 nm, and 20 nm mesoporous silica are significantly inhibited, and the 14 nm group shows the strongest ability to inhibit adhesion behavior of macrophages.

**Figure 6:**
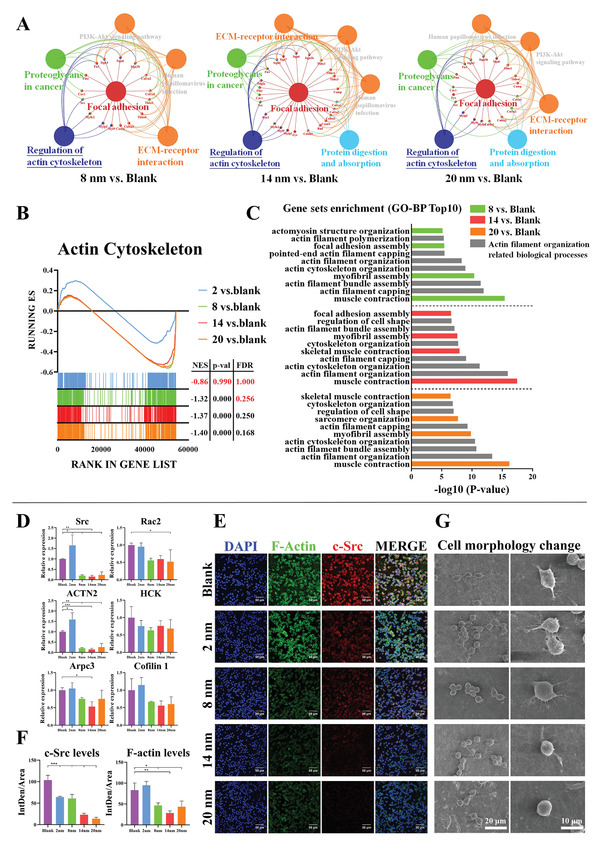
The adhesion‐mediated actin cytoskeleton assembly of macrophages is inhibited on generated fibrin network with thinner fibers. A) The functional enrichment analysis of adhesion downregulated genes from 8, 14, and 20 nm versus Blank using ClueGO, a plug‐in of Cytoscape. B) GSEA reveals the global difference in 2, 8, 14, and 20 nm versus Blank of actin cytoskeleton of GO cellular component. |NES| > 1, NOM *p*‐val < 0.05 and FDR *q*‐val < 0.25 are considered significant. C) Bar diagram shows the Top10 biological process enrichment items of downregulated actin cytoskeleton key genes from 8, 14, and 20 nm versus Blank. The gray items represent actin filament organization related biological processes. D) RT‐qPCR results of actin filament assembly related gene expressions in macrophages cultured on fibrin network. E,F) Representative immunofluorescence images and semi‐quantitative statistical analysis of F‐actin (assembled filamentous actin) and c‐Src (one of key signaling molecules regulating F‐actin assembly) in macrophages cultured on fibrin network. G) Representative SEM images of cell morphology change of macrophages cultured on fibrin network. Data are presented as means ± s.d.; *n* = 3; *p < 0.05, **p < 0.01, ***p < 0.001 by one‐way ANOVA with Tukey's post hoc test.

After confirming the vital role of pore size on cell adhesion of macrophages, we analyze the changes in intracellular functional events mediated by cell adhesion. We find that the selected downregulated adhesion genes from the 8 nm, 14 nm, and 20 nm groups affect the regulation of actin cytoskeleton (**Figure** **6**A,B, Figure S6A, Supporting Information), which can be related to the limited assembly of actin filaments (Figure 6C, Figure S6B, Supporting Information). Genes involved in actin filament assembly‐related events are then ruled out. We find that the inhibition of actin filament assembly by the 14 nm group appear to be the most significant, followed by that in the 20 nm and 8 nm groups; this is consistent with the adhesion behavior of these three groups (Figure S6C,D, Supporting Information).

To verify the downregulation of genes involved in actin filament, some of the identified genes (Src, Rac2, HCK, ACTN2, Arpc3, and Cofilin1) are detected by RT‐qPCR. It is shown that these genes are downregulated in the 8 nm, 14 nm, and 20 nm groups (Figure 6D). To further confirm these RT‐qPCR‐based analyses, c‐Src is selected for immunofluorescent staining analyses, and it is found that expression of c‐Src is indeed suppressed (Figure 6E,F). Since the assembled filamentous actin (F‐actin) is the endpoint regulated by these cytoskeleton‐related genes, we perform co‐staining of F‐actin and phalloidin, and find that the assembly levels of F‐actin are downregulated in the 8 nm, 14 nm, and 20 nm groups (Figure 6E,F). Since different assembly levels of F‐actin may lead to change in cell morphology, such as the podosome formation, SEM imaging is used to observe impact of assembly levels of F‐actin on macrophage morphology. It is shown that macrophages in the 2 nm, 8 nm, and blank groups tend to form a more elongated shape, while macrophages in the 14 nm and 20 nm groups maintain a round shape with less podosome formation (Figure 6G). Thus, these pieces of data further confirm that the actin filament assembly in the 14 nm and 20 nm groups are less active.

**Figure 7:**
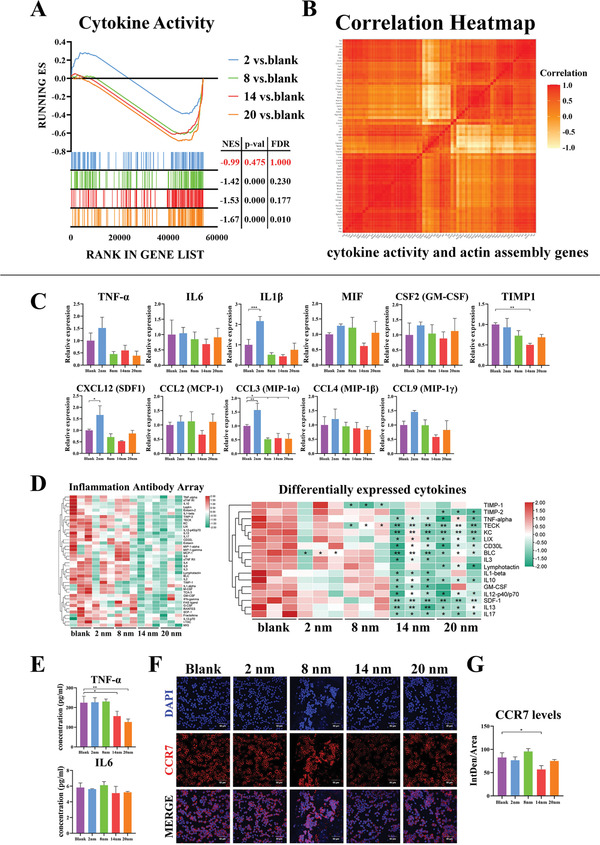
The pro‐inflammatory response associated with actin cytoskeleton assembly of macrophages is alleviated on generated fibrin network with thinner fibers. A) GSEA reveals the global difference in 2, 8, 14, and 20 nm versus Blank of cytokine activity of GO molecular function. |NES| > 1, NOM *p*‐val < 0.05 and FDR *q*‐val < 0.25 are considered significant. B) Pearson correlation analysis of downregulated actin filament assembly genes and downregulated cytokine activity related genes. C) RT‐qPCR results of pro‐inflammatory cytokines and chemokines related gene expressions in macrophages cultured on fibrin network. D) Cytokine profiles of cell cultured supernatant of macrophages cultured on fibrin network using Inflammation Antibody Array. E) TNF‐*α* and IL6 concentration of cell cultured supernatant of macrophages cultured on fibrin network using ELISA. F,G) Representative immunofluorescence images and semi‐quantitative statistical analysis of CCR7 (classic M1‐type macrophage marker) in macrophages cultured on fibrin network. Data are presented as means ± s.d.; *n* = 3; *p < 0.05, **p < 0.01, ***p < 0.001 by one‐way ANOVA with Tukey's *post hoc* test.

It has been reported that the actin filament assembly of macrophages is closely related with their inflammatory responses,^[^ ^34^ ^]^ and the pro‐inflammatory responses of macrophages are suppressed when actin filament assembly is inhibited.^[^ ^35^ ^]^ Given that reduced adhesion lead to inhibition of actin filament assembly, we investigate whether mesopore sizes might regulate the inflammatory responses of macrophages. We find that a number of pro‐inflammatory cytokines (TNF‐*α*, IL‐6, IL‐18, LTB, and MIF) and chemokines (CCL3, CCL4, CCL9, CCL2, CXCL12, CXCL14, CXCL2, and IL‐16) genes are downregulated in the 8, 14, and 20 nm groups from the RNA‐seq results (**Figure** **7**A, Figure S7A,B, Supporting Information). Most downregulated cytokines are found similarly in the 8, 14, and 20 nm groups, suggesting that they display similar inhibited pro‐inflammatory responses (Figure S7C, Supporting Information). These downregulated pro‐inflammatory cytokine and chemokine genes are highly correlated with the screened actin assembly‐related genes, which indicate that the inhibition of actin filament assembly is closely related with the downregulated pro‐inflammatory responses of macrophages in thinner fibers (Figure 7B).

To further confirm whether the inflammatory responses of macrophages are downregulated, mRNA of pro‐inflammatory cytokines (TNF‐*α*, IL‐6, IL‐1*β*, MIF, and GM‐CSF) and chemokines (CXCL12, CCL2, CCL3, CCL4, and CCL9) are detected by RT‐qPCR. It is shown that most cytokines are downregulated, with the 14 nm group as the most prominent for their ability to repress expression of pro‐inflammatory cytokines (Figure 7C). In consistent with RT‐qPCR‐based mRNA analyses, cytokine array and ELISA both confirm that classic pro‐inflammatory cytokines and chemokines, such as TNF‐*α*, IL‐1*β*, IL‐12, and SDF‐1, are downregulated in the 14 and 20 nm groups (Figure 7D,E). It is also shown that there is the least expression of CCR7, a representative macropahge M1‐type marker in the 14 nm group, suggesting the inhibition of M1 macrophage polarization in the 14 nm group (Figure 7F,G).

In summary, these results confirm that when the pore size of mesoporous silica is larger than 9 nm, the fibrin network is thinner, and the contact area between cells and the fibrin network is smaller, thereby inhibiting the cell adhesion of macrophages. This leads to the suppression of actin filaments and the inhibition of inflammation. It should be noted that some of these effects are also observed in the 8 nm group, which might be attributed to the difficulty in maintaining a completely uniform pore size during the preparation of materials. The average pore size obtained by BET is not equal to the absolute uniform pore size (absolute uniformity is also impossible). The 8 nm mesoporous particles may also have pore sizes greater than 9 nm that can adsorb fibrinogen, leading to a similar regulation effect as in the 14 and 20 nm groups. This implies that future study could focus on the fabrication of mesoporous materials with homogeneous large pore size, to meet the precision manipulation of clot‐immune complex.

### 2.5 Implications for the Development of Advanced Clot‐Immune Regulatory Materials

As mentioned previously, blood clot formation and inflammatory responses are two closely interactive consecutive events.^[^ ^5^ ^]^ As an important initial event in the entire regenerative or reparative process, this clot‐immune complex determines the eventual outcome. However, most of the current development strategies for regenerative or reparative materials are aimed at directly regulate the end‐point functional cells, while ignore the starting events at an early stage. This goes against the natural regenerative or reparative rule that starting events determine the subsequent consecutive events. This could lead to some unexpected in vitro and in vivo results and a low clinical translation rate. Therefore, it is still of great importance to develop clot‐immune regulatory materials for improving regenerative or reparative outcomes.

**Figure 8:**
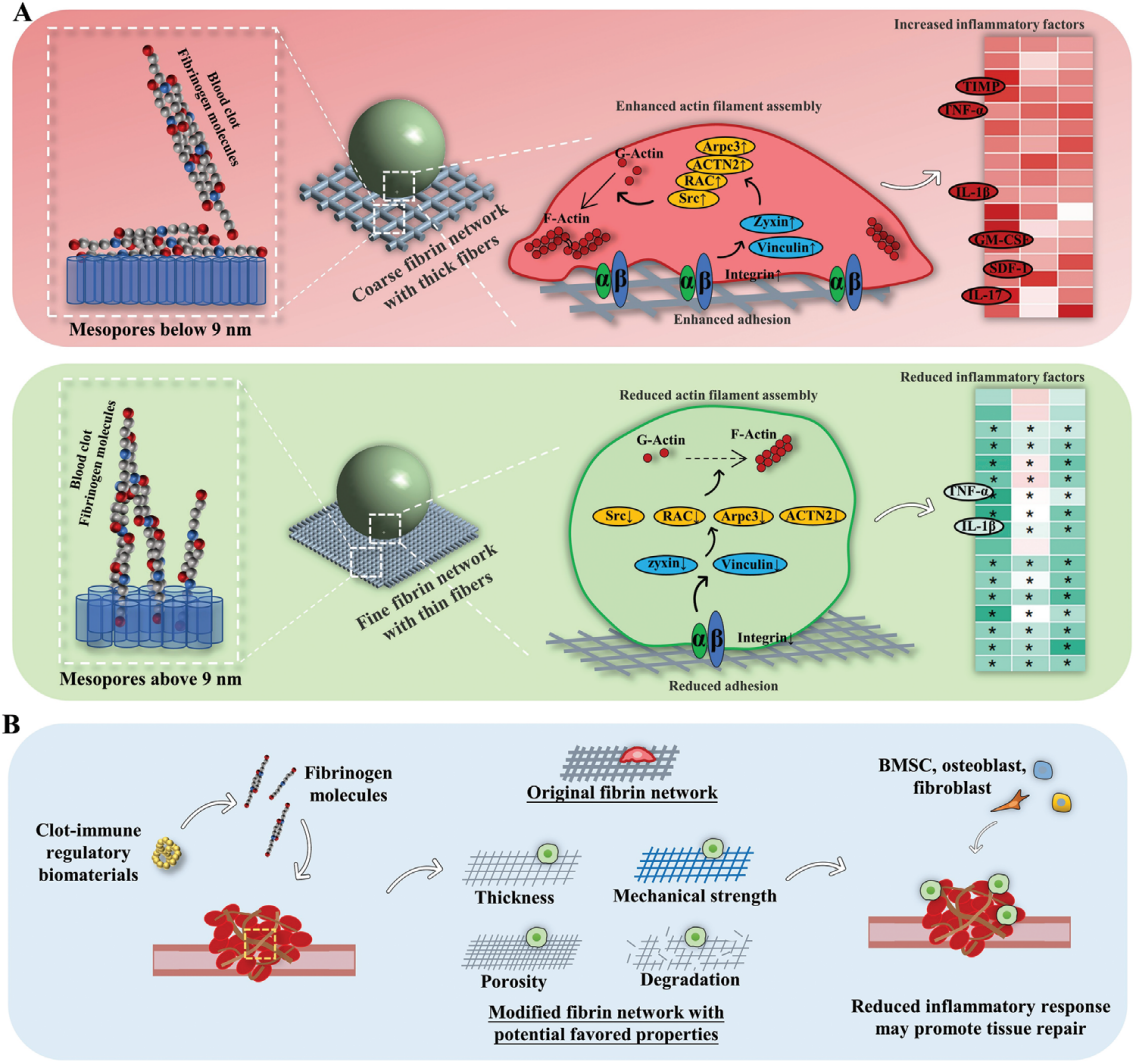
Schematic figure of the underlying mechanisms of mesopore size regulating clot‐immune responses and the strategies to develop clot‐immune regulatory biomaterials. A) Mesopore size below 9 nm has little impact on fibrin formation and the following biological activities of cell. Mesopore size above 9 nm induces thinner fibers and smaller contact area with cells, which reduces inflammatory response via pathway of the cell adhesion‐cytoskeleton assembly‐inflammatory response. B) The development strategy diagram of clot‐immune regulatory materials by tuning fibrin network parameters including fiber thickness, fibrin porosity, density, mechanical strength, and degradation pattern.

Although regulating the clot‐immune complex was a difficult process, we succeeded in regulating this complex by targeting fibrin network changes (**Figure** **8**A). This implies the feasibility of the strategy of tuning fibrin network parameters for the development of clot‐immune regulatory materials. Blood clot fibrin has a series of parameters that may be involved in the responses of clot‐immune complex, including fiber thickness, fibrin porosity, density, mechanical strength, and degradation pattern (Figure 8B). The current strategy uses fiber thickness as the regulation target and regulated the induced inflammatory response. However, the change in fiber thickness is usually accompanied by a series of other changes (porosity, mechanical strength, degradation, etc.). For example, mesopore may bi‐regulate the fiber thickness and the density of fibrin network.^[^ ^36^ ^]^ This thickness‐dense coupling regulation of fibrin network can become an effective strategy to manipulate the balance between inflammation and osteogenesis.

In addition, the present study uses the same concentration of mesoporous silica to avoid potential coagulation effects in terms of concentrations. However, the higher the material concentration is, the more additional nuclei may be formed thus inducing faster coagulation rate. This implies that the concentration of mesoporous silica may be involved in the coagulation regulation. How to adjust the concentration of mesoporous silica to regulate coagulation is an interesting topic, which may provide another improved aspect for the manipulating of coagulation via the coupling regulation of mesopore size and particle concentration. Furthermore, complex in vivo environment is different from the in vitro one. In vivo study may be required to further understand the regulatory effects of mesopore on the fibrin network.

## 3 Conclusion

Fibrin formation and blood clotting can be manipulated by tuning of the mesopore size of mesoporous materials. Mesopore sizes of 2 and 8 nm provide little or no exogenous nuclei and have no observable effect on regulating fibrin formation. In contrast, mesopore sizes of 14 and 20 nm can enrich fibrinogen molecules and form exogenous nuclei. With additional nuclei, thinner fibers can be generated by reducing the extent of lateral aggregation. These thinner fibers eventually regulate cell adhesion and cytoskeleton assembly and induce a milder inflammatory response in macrophages. Thus, advanced clot‐immune regulatory materials may be developed by regulating fibrin formation with fine‐tuned mesopore sizes.

## 4 Experimental Section

### Mesoporous Silica Preparation

Tetraethyl orthosilicate (10 mL), absolute ethanol (95 mL), ultrapure water (10 mL), and hydrochloric acid solution (1 mol L^–1^, 10 µL) were mixed, stirred, and hydrolyzed for 4 h to obtain the silicate precursor solution. The pH value of the solution was adjusted to 6.6, 7.4, 7.8, and 8.0, respectively. The solution was sealed and kept at 25 °C for 24 h to gel. After gelation, *n*‐hexane solvent (100 mL) was added to groups with pH 6.6, 7.8, and 8.0 for solvent replacement. Hexamethyldisilazane was added for modification. The modified rubber block and unmodified rubber block with a pH of 7.4 were cleaned with absolute ethanol and mixed with absolute ethanol at a ratio of 1:2. A cell disruption‐grade probe‐type ultrasonic pulverizer was used to disperse the mixed solution. After dispersal, the solution was placed in a 70 °C oven for 12 h and burned in a muffler oven at 600 °C. After cooling, the nanoparticle powders were collected and prepared for the subsequent experiments.

### Physicochemical Characterization

The porosity and specific surface area were tested using a nitrogen adsorption and desorption instrument (Mike ASAP2460, USA). The particle size and zeta potential were tested using a nanoparticle size and zeta potential analyzer (Malvern Zetasizer Nano‐ZS90, UK) after grinding and ultrasonic dispersion. The morphological characteristics of the samples were observed using a field‐emission SEM (Gemini500, Germany) with a low voltage of 0.5 kV. Integration mode without gold spraying was used to prevent electron irradiation damage (Figure S7, Supporting Information). An FTIR microscope (Nicolet 6700, USA) was used at a wavelength of 400–4000 cm^–1^. Samples were mixed with potassium bromide and pressed into the tablet. An X‐ray powder diffractometer (Empyrean, Netherlands) was used to analyze the conformational composition. Uncalcined sample particles were placed in a crucible for synchronous thermal analysis using an asynchronous thermal analyzer (STA449F3/Nicolet 6700, Germany). The samples were analyzed in air atmosphere with a heating rate of 10 °C min^–1^ and a temperature range from ambient temperature to 900 °C.

### Cell Culture

RAW 264.7 cell line was selected as the experimental cell. Dulbecco's Modified Eagle Medium (DMEM; Gibco, USA) supplemented with 10% fetal bovine serum (Gibco) and 1% penicillin/streptomycin (Gibco) was used as the complete medium. Culture flasks were placed in a cell incubator (37 °C, 5% CO_2_) and the medium was replaced every two days. The cells were passaged after 80% convergence.

### Cytotoxicity Assay

For each particle with a certain pore size, four sub‐groups were set depending on the particle concentration (100, 1000, 3000, and 5000 µg mL^–1^). The conditioned medium was harvested after immersion with the particle for 24 h. Five duplicates were tested. Cells were seeded on plates overnight and the medium was replaced with the conditioned medium. Cell cytotoxicity tests were assessed using the Cell Counting Kit‐8 (CCK‐8; Dojindo, Japan) after 1, 3, and 5 days.

According to the results of CCK‐8 assay, the concentration of mesoporous silica used in the following biological experiments was set to 1000 µg mL^−1^. For the following blood/blood plasma interaction studies, construction of fibrin gel, and blood clot fibrin model, the mother liquor of mesoporous silica in 10 mg mL^−1^ was prepared and mixed with blood/blood plasma in a volume ratio of 1:10, thus obtaining the final concentration of 1000 µg mL^−1^ of mesoporous silica.

### Whole Blood and Blood Plasma Preparation

All experimental protocols were reviewed by the Experimental Animal Ethics Committee of Sun Yat‐sen University, and conformed with the principles of animal protection, animal welfare and ethics (Approval NO: SYSU‐IACUC‐2020‐000484). Six‐eight‐week‐old male SD rats (purchased from the Laboratory Animal Center of Sun Yat‐Sen University) were used. The rats were anesthetized by intramuscular injection with tiletamine and zolazepam solution (Zoletil, France). Laparotomy was performed, and the septum was separated upward to expose the apex of the heart. Whole blood was collected. For blood plasma preparation, whole blood was immediately transferred to an anticoagulant tube followed by centrifugation at 3000 rpm for 10 min. Blood plasma was collected by carefully aspirating the supernatant.

### Clotting Time Test In Vitro and Hemostasis In Vivo

To verify the coagulation function of mesopores, clotting time test in vitro and hemostasis in vivo were conducted. The clotting time was recorded when a complete blood clot was formed without flow. Hemostasis in vivo was tested using a rat liver injury model.^[^ ^37^ ^]^ The experimental rats were anesthetized. A midline laparotomy was carefully performed to expose the liver. Gauze was placed under the left inner lobe to absorb the blood, and the lower 1/3 of the lobe was excised with tissue scissors. For the experimental groups, the injury site was immediately covered with particles (15 mg). For the blank group, no material was placed. The coagulation time was recorded as soon as the hemorrhage stopped. Each group had three biological replicates.

### Water Absorption of Mesoporous Silica and Resulting Hemorheology

The water absorption property of mesoporous silica is one of the causes of the reduced coagulation time via a blood concentrating effect.^[^ ^25^ ^]^ The water absorption ability of mesoporous silica and its effect on hemorheology were detected. For the water absorption test, 20 mg of mesoporous silica were immersed in 1 mL of ultrapure water for 1 min, followed by centrifugation at 8000 rpm for 2 min. The supernatant was carefully removed and weighed. Water absorption ability was calculated as the increased weight divided by the weight of the particles. For the hemorheology test, 20 nm mesoporous silica was mixed with rat blood in a heparin sodium anticoagulant tube. The specific volume of red blood cells and whole blood viscosity were measured using a blood viscometer (Sysmex, Japan). Each group had three biological replicates.

### Protein Adsorption of Mesoporous Silica and Resulting Clotting Time Test

Protein adsorption property is another important mechanism for reducing coagulation time.^[^ ^26^ ^]^ To determine the change in protein concentration of the plasma after mesoporous silica adsorption, mesoporous silica was mixed with plasma and centrifuged at 1000 rpm for 1 min. The protein concentration in supernatant was detected by BCA protein assay following the manufacturer's instruction. To determine the amount of protein adsorbed by the material, the blood plasma was diluted 20 times with ultrapure water. The BCA protein assay was repeated to measure the protein concentration in the supernatant. To explore the protein distribution, the pore volume change was measured before and after protein adsorption using the nitrogen adsorption and desorption instrument. Finally, the clotting time test was repeated using the BSA‐pretreated particles.

### Potential Key Coagulation Factors Detection

To identify key coagulation factors, the concentration of coagulation factors was screened after mesoporous silica adsorption, and the aPTT, PT, TT, and INR were measured. For the screening test, mesoporous silica was mixed with blood plasma followed by centrifugation at 1000 rpm for 1 min. The supernatant was tested with a coagulation analyzer (CS5100, Japan) to screen the coagulation factors, followed by aPTT/PT/TT/INR measurement. To quantitatively evaluate the adsorption capacity of fibrinogen, a rat fibrinogen ELISA Kit (CUSABIO, China) was used to measure the fibrinogen concentration in the supernatant according to the manufacturer's instructions.

### Computer Simulation of Mesopore‐Mediated Fibrin Assembly

To verify the mesopore‐mediated fibrin formation, built a coarse‐grained model of fibrinogen was built. The human fibrinogen (PDB ID: 3GHG) all‐atom molecule was divided into nine beads based on the shape‐based coarse‐graining approach.^[^ ^38^, ^39^ ^]^ The positions of the beads and their masses were estimated by a topology network algorithm.^[^ ^40^ ^]^ For the sake of stability, *σ* = 2 nm was selected, and the cutoff radius was 5*σ* to allow interactions between distant, sparsely dispersed fibrinogen molecules. The 825 CGMD fibrinogen model was introduced to a 300 nm × 300 nm × 200 nm periodic box at a concentration of 4.5 g L^–1^. The density of the fibrinogen molecules in this equilibrated system represents the physiological concentration of human fibrinogen,^[^ ^41^ ^]^ which is in the range of 3–4.5 g L^–1^. To investigate the effect of mesoporous silica, a porous substrate (200 nm × 200 nm × 90 nm) with a 20 nm hole was inserted into the system. The interaction parameters for water beads were held constant, as they were already parameterized.^[^ ^37^ ^]^ Numerical integration was performed using the velocity Verlet scheme,^[^ ^42^ ^]^ and the temperature of the system was controlled using a velocity‐scaling thermostat. All CGMD simulations in this work were performed using LAMMPS.^[^ ^43^ ^]^

### Fibrin Network Characterization

After revealing the adsorption properties of fibrinogen on mesoporous silica, a fibrin gel model was established to determine the effect of fibrinogen adsorption on the characteristics of fibrin network. To prepare the fibrin gel model, mesoporous silica was mixed with blood plasma and thrombin reagent (Haifei, China) and left for 10 min to form a fibrin gel. The blank group used ultrapure water instead of a mesoporous silica solution. For morphological observation, the fibrin network was soaked in a 2.5% glutaraldehyde solution for fixation, followed by dehydration with graded ethanol. Solvent replacement and lyophilization were then performed. The morphological characteristics of the samples were observed by SEM. SEM‐EDS was used to detect the silicon element distribution. The porosity and fibrin diameter of the fibrin network were calculated using ImageJ software. An atomic force microscope (AFM, Dimension Fastscan, Germany) was used to test the elastic modulus of the fibrin network. A thermogravimetric analyzer (STA449F3/Nicolet 6700, Germany) was used to detect the amount of particles in the fibrin network. The samples were analyzed in air atmosphere with a heating rate of 10 °C min^–1^ and a temperature range from ambient temperature to 900 °C. Each group had three biological replicates.

### Blood Clot Fibrin Characterization

The changes in the fibrin network of a blood clot were explored. The blood clot was prepared by mixing mesoporous silica and rat blood. After 2 h, the resulting blood clots were collected for morphological observation, mechanical testing, and AFM analysis. For the morphological observation, the clots were soaked in a 2.5% glutaraldehyde solution for fixation. Dehydration was performed using graded ethanol, followed by solvent replacement and lyophilization. The morphological characteristics of the samples were then observed by SEM. The porosity and fibrin diameter of the fibrin network were calculated using ImageJ software. For the mechanical test, a universal mechanical testing machine (Instron E3000, UK) was used to evaluate the elastic modulus of the clot. For the detection of the micro elastic modulus, the fibrin network on the blood clot was carefully isolated as a fibrin network model described below. AFM was used to detect the elastic modulus of the fibrin network. Each group had three biological replicates.

### Finite Element Analysis of Cell–Fibrin Network Interaction

To demonstrate the interaction between the fibrin network and cell, finite element analysis was used to simulate the contact of cells on the fibrin network by DesignModeler (ANSYS 19.2, USA). Two types of fibrin networks were constructed: a coarse fibrin network model and a fine fibrin network model. For the coarse fibrin network model, the fiber diameter and fibrin spacing were set as 1 µm and 3 µm, respectively. For the fine fibrin network model, the fiber diameter and fibrin spacing were set as 0.5 µm and 0.75 µm, respectively. A sphere was selected as the cell model, and the cell diameter was set as 12 µm. It was assumed that all the tissues of the model were continuous, homogeneous, and isotropic linear elastic materials. The elastic modulus of the fibrin network was set as 0.001 MPa, and the Poisson's ratio was set as 0.49. The bottom surface of the model was given a rigid constraint with full degrees‐of‐freedom to prevent the displacement of the network. To better reflect the contact between the cell and fibrin network, the cell was set as a rigid sphere, and the deformation area between the cell and network was simulated under the condition of gravity. The image of the deformation area of the fibrin network was imported into ImageJ to calculate the deformation area. The cell was further set as an elastic sphere, the elastic modulus was set as 0.001 MPa, and the Poisson's ratio was set as 0.49. The deformation area of the cell and fibrin network was simulated, and the deformation area was calculated under the condition of gravity.

### Cell–Fibrin Network Model Construction

To construct the cell–fibrin network model, the fibrin network on the blood clot was extracted as follows. The blood clot mixed with mesoporous silica solution was punched with hole followed by the careful removal of the marginal part. The residual central part was then treated with erythroblast lysate buffer. The buffer was replaced every 12 h and lasted for two days. The fibrin network was obtained after washing three times in PBS (Figure S8, Supporting Information). To investigate the effect of silicon ion on macrophages, extracts from blood clot fibrin model was collected for the detection of silicon ion concentration using inductively coupled plasma atomic emission spectrometer (ICP‐AES, Agilent, China).

Cells were seeded on the generated fibrin network and cultured for 48 h. The cells were then subjected to RNA‐seq, RT‐qPCR and immunofluorescence staining. The cell culture supernatants were collected for cytokine array and ELISA test.

### RNA‐Seq

The total RNA of cells on fibrin network was extracted using the TRIzol reagent (Beyotime, China). Paired‐end libraries were synthesized using the TruSeq RNA Sample Preparation Kit (Illumina, USA) following the TruSeq RNA Sample Preparation Guide. Purified libraries were quantified by the Qubit 2.0 Fluorometer (Life Technologies, USA) and validated by the Agilent 2100 bioanalyzer (Agilent Technologies, USA) to confirm the insert size and calculate the mole concentration. Clusters were generated by cBot with the library diluted to 10 × 10^−12^ m, and then sequenced on the Illumina NovaSeq 6000 (Illumina, USA). The library construction and sequencing were performed by Sinotech Genomics Co., Ltd (Shanghai, China).

### Bioinformatic Analysis

Paired‐end sequence files (fastq) were mapped to the reference genome using Hisat2 (v2.0.5). Gene abundance was expressed as fragments per kilobase of exon per million reads mapped (FPKM). Stringtie software was used to count the fragments within each gene. The TMM algorithm was used for normalization. Differential expression analysis for mRNA was performed using the R package DESeq2. Differentially expressed RNAs with |log2(FC)| >1 and adjusted *p*‐value < 0.05, considered as significantly modulated, were selected to further analysis. GO (Gene Ontology) analysis of biological processes, cellular components, and molecular function; and KEGG pathway analysis (Kyoto Encyclopedia of Genes and Genomes) were performed via DAVID Bioinformatics Resources (v6.8). GSEA (v4.1.0) was used to analyze the differences between two groups in a predefined gene set and to select the leading‐edge subset contributing to the differences. ClueGO (v2.5.8), a Cytoscape plug‐in, was used for further enrichment analysis and visualization. All data visualization was performed using GraphPad Prism (v8.0.1), TBtools (v1.095), OmicStudio tools (https://www.omicstudio.cn/tool), and Hiplot Platform (https://hiplot.com.cn).

### RT‐qPCR

The total RNA of cells was extracted as described in *RNA‐seq*. Reverse transcription to cDNA was performed with the cDNA Synthesis SuperMix (YEASEN, China). The SYBR Green Master Mix (YEASEN, China) was used to perform RT‐qPCR. The primers of target genes (Table S2, Supporting Information) were designed according to the cDNA sequences in NCBI and verified by BLAST databases. cDNA, primer and probe mix were prepared and RT‐qPCR were performed using Applied Biosystems QuantStudio 7 Flex (Life Technologies, USA). The endogenous control was the housekeeping gene GAPDH and the analysis results of RT‐qPCR were obtained using comparative CT method (ΔΔCt). Each group had three biological replicates.

### Immunofluorescence Staining

The cells on fibrin network were fixed with 4% paraformaldehyde (Beyotime, China) and permeated with 0.2% TritonX‐100 (Sigma‐Aldrich, USA). Cells were blocked with 1% BSA in PBS and incubated with Integrin *β*3 (Affinity, AF6086, 1:100), Zyxin (Abcam, ab109316, 1:500), c‐Src (Proteintech, 60315‐1‐Ig, 1:100), and CCR7 (Abcam, ab32527, 1:100) antibody overnight at 4 °C. After incubation, the cells were washed and incubated with Alexa Fluor 647 goat anti‐rabbit secondary antibody (Beyotime, China) and Alexa Fluor 594 goat anti‐mouse secondary antibody (Emarbio, China) at 20 °C for 1 h. Then the cells were washed and stained for F‐actin (Beyotime, China) and nuclei with DAPI staining solution (Beyotime, China). Fluorescent images were observed with a confocal laser scanning microscope (FV3000 Olympus, Japan).

### Cytokine Array

The cell‐cultured medium was collected and centrifuged (10 000 rpm, 5 min, 4 °C) to obtain the supernatant. An uncultured media sample was tested as the negative control. The cytokine profiles were analyzed using the Mouse Inflammation Antibody Array C1 (AAM‐INF‐1, RayBiotech, USA). The exposure intensity was detected using a chemiluminescence imaging system (ChemiDoc, Bio‐Rad, USA). The signal intensity was extracted using ImageJ software, and the relative protein levels were quantified using the Excel‐based analysis software tools offered by RayBiotech.

### ELISA

The cell culture supernatant was collected as described in *Cytokine array*. ELISA were performed using a mouse TNF‐*α* ELISA Kit (Boster, China) and a mouse IL‐6 ELISA Kit (CUSABIO, China).

### Statistical Analysis

Each experiment was repeated at least three times independently and the sample size (*n*) was presented in each figure legend. The presented SEM micrographs and immunofluorescence micrographs were representative. All the experimental results were expressed as means ± standard deviations (s.d.). For data analysis, unpaired two‐tailed Student's *t*‐test was carried out when comparing the means of two groups. When comparing data from more than two groups, one‐way (for one independent variable) or two‐way (for two independent variables) analysis of variance (ANOVA) followed by Tukey's multiple comparison *post hoc* test was carried out. All statistical methods were described in each figure legend. Statistical analysis of data was performed using GraphPad Prism (v 8.0.1). In all cases, *p*‐value below 0.05 was considered statistically significant (*p < 0.05; **p < 0.01; ***p < 0.001).

## Conflict of Interest

The authors declare no conflict of interest.

## Supporting information

Supporting Information

## Data Availability

Research data are not shared.
