# Supplementary material for: Mesopore Controls the Responses of Blood Clot‐Immune Complex via Modulating Fibrin Network
Source: Adv Sci (Weinh). 2021 Nov 24;9(3):2103608. doi: 10.1002/advs.202103608 (PMC8787416; doi:10.1002/advs.202103608)
Supplement: Supplementary file 1 — Supporting Information [file ADVS-9-2103608-s001.pdf]

Supporting Information

for *Adv. Sci.*, DOI: 10.1002/advs.202103608

Mesopore controls blood clot-immune complex responses via modulating fibrin network formation

Shiyu Wu, Zhengjie Shan, Lv Xie, Mengxi Su, Peisheng Zeng, Peina Huang, Lingchan Zeng, Xinyue Sheng, Zhipeng Li, Gucheng Zeng, Zhuofan Chen*, and Zetao Chen**

Supporting Information

Mesopore controls blood clot-immune complex responses via modulating fibrin network formation

Shiyu Wu, Zhengjie Shan, Lv Xie, Mengxi Su, Peisheng Zeng, Peina Huang, Lingchan Zeng, Xinyue Sheng, Zhipeng Li, Gucheng Zeng, Zhuofan Chen*, and Zetao Chen**

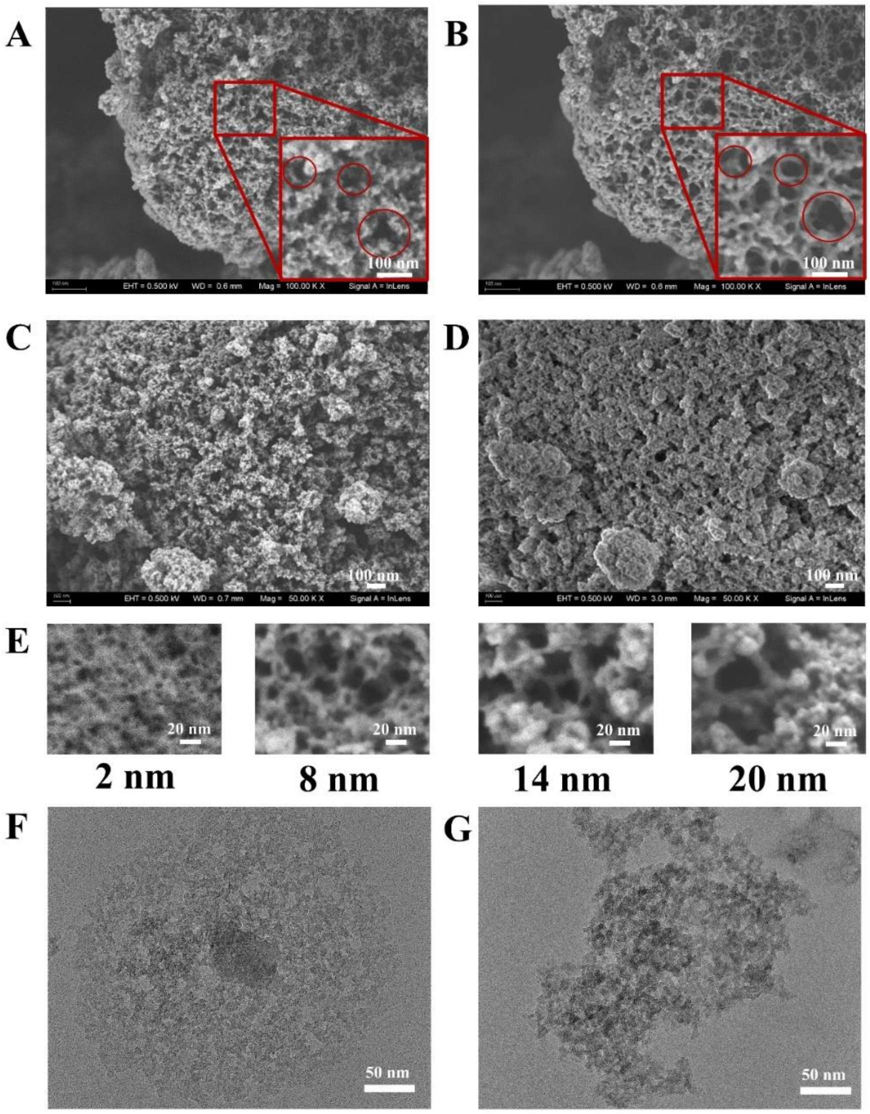

Figure S1. The optimization of SEM detection parameters. (A) SEM image of electron beam contacts with mesoporous silica instantly. (B) SEM image of electron beam contacts with mesoporous silica after 10 seconds, the original morphology of mesoporous silica is destroyed by electron beam, indicating the necessity of avoiding delayed photography (red circles). (C) SEM image in integration mode shows intact structure of mesoporous silica. (D) SEM image in fast scan mode, the original morphology of mesoporous silica is destroyed by charging, indicating the necessity of using integration mode. (E) SEM images of mesopores with different pore diameters under integration mode. (F) The mesoporous silica is dispersed by the electron beam of TEM with unclear mesopore structure. (G) The mesopore structure is damaged by the electron beam of TEM.

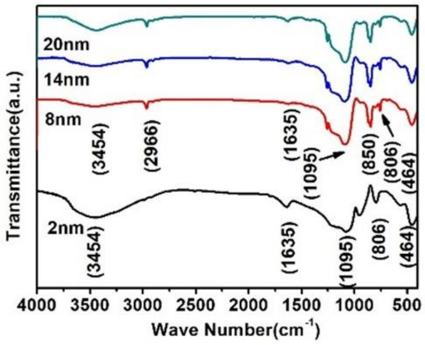

Figure S2. Fourier transform infrared spectrum (FTIR) analysis of mesoporous silica without heat treatment. The prepared mesoporous silica before heat treatment contains methyl groups.

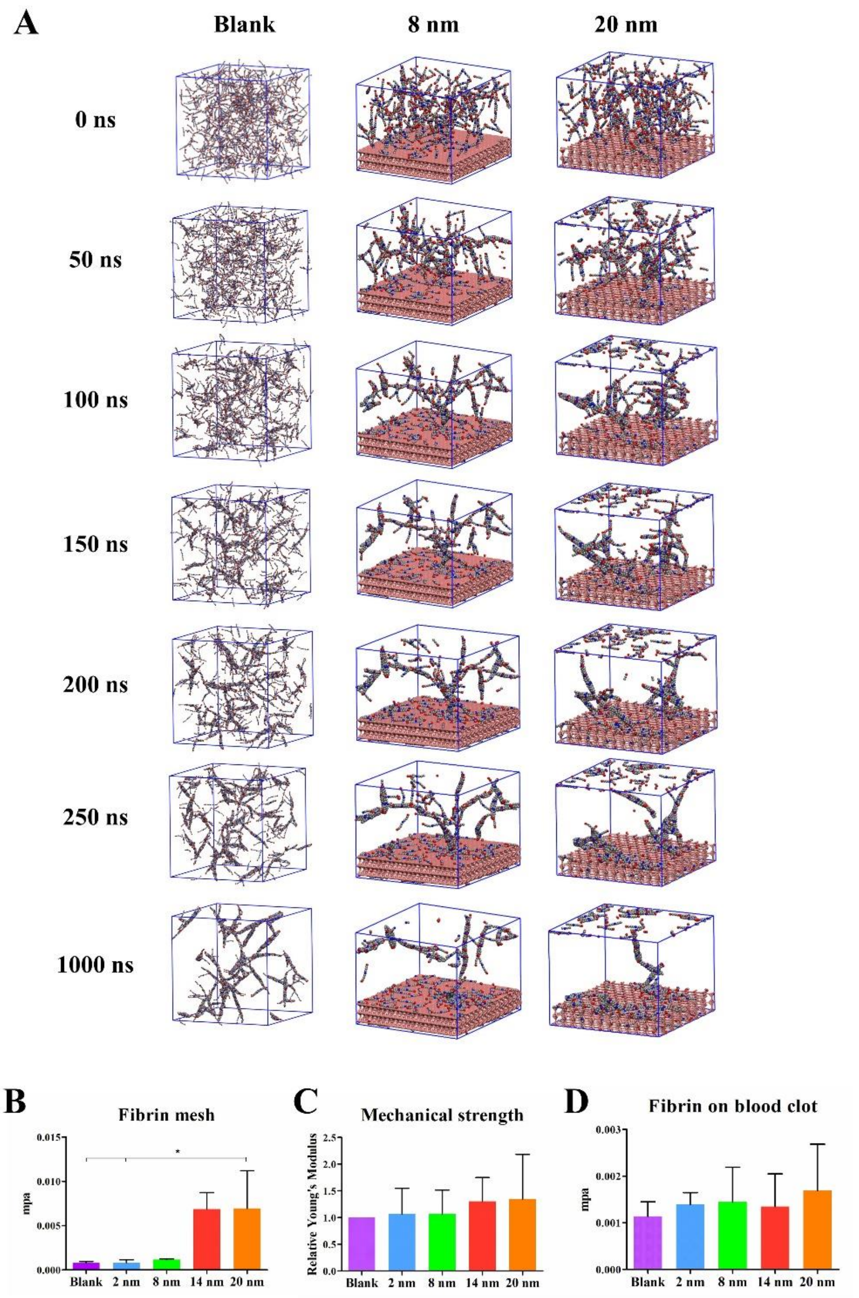

Figure S3. The molecular dynamics simulation of fibrinogen polymerization from 0-1000 ns and the mechanical properties of the generated fibrin network. (A) Molecular dynamics simulation images of the 8 nm, 20 nm, and blank groups at 0, 50, 100, 150, 200, 250, and 1000 ns, respectively. (B) Micromechanical strength of extracted fibrin network. (C) Mechanical strength of mesoporous silica-mediated blood clots. (D) Micromechanical strength of fibrin network on blood clots. Data are

presented as means ± s.d.; $n = 3$; *$p < 0.05$ by one-way ANOVA with Tukey's *post hoc* test.

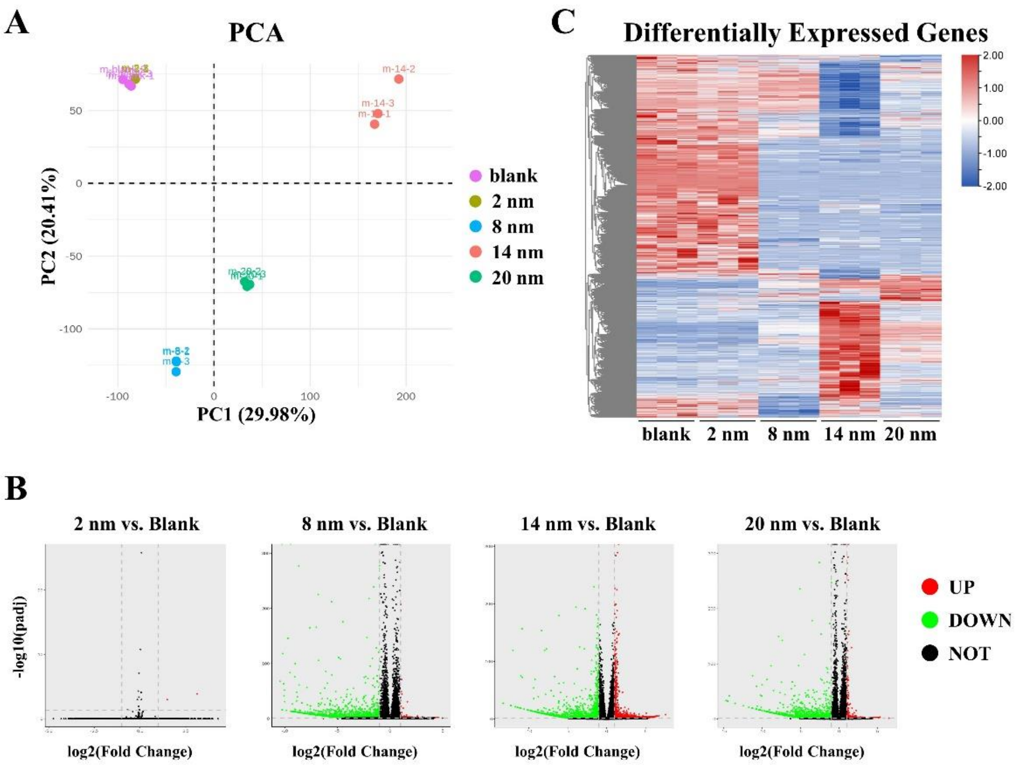

Figure S4. The quality evaluation and differential analysis of RNA-seq. (A) The principal component analysis (PCA) reveals the inter-group differences and intra-group repeatability (PC1=29.98%, PC2=20.41%). (B) Volcano maps show the differentially expressed genes of each group versus blank (|log2(FC)| value > 1 and adjusted p-value < 0.05). (C) Heatmap shows the gene expression pattern of differentially expressed genes in each group vs. blank. Tile color, log2 (FPKM).

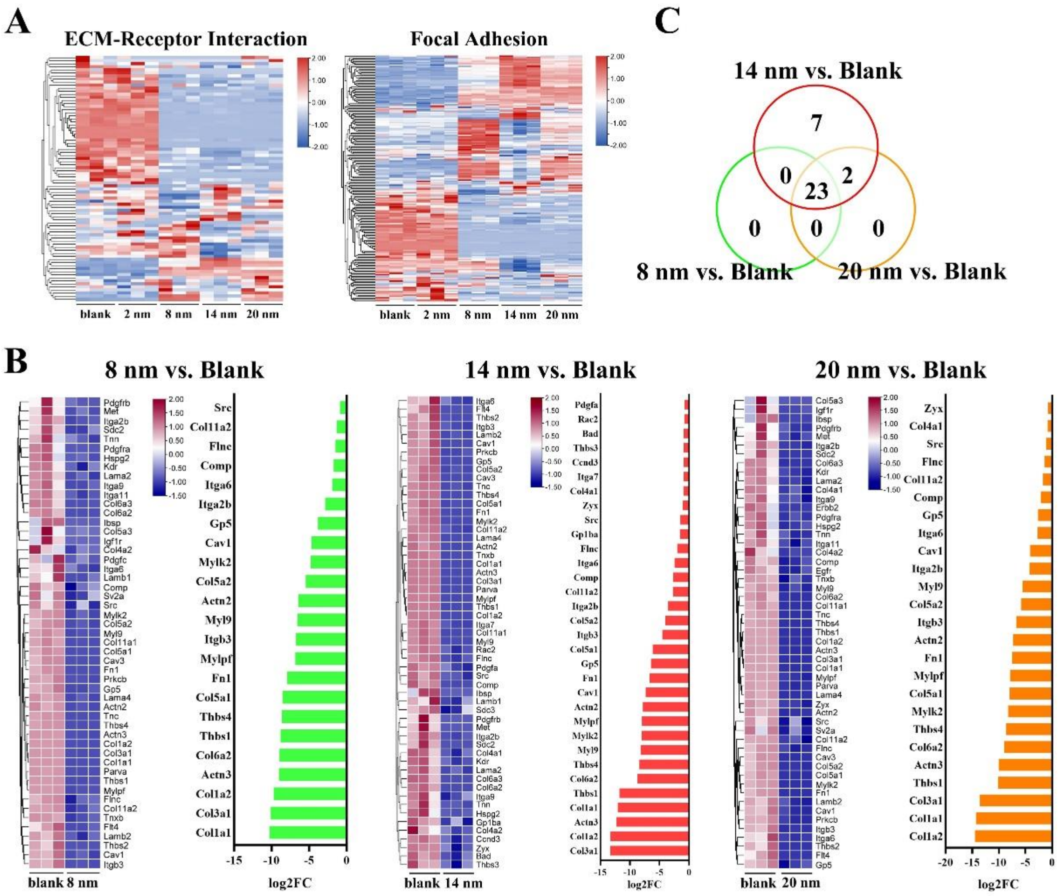

Figure S5. The expression differences of adhesion related genes in 2 nm, 8 nm, 14 nm, 20 nm vs. blank. (A) Heatmaps show all genes expression from ECM-Receptor Interaction and Focal Adhesion. Tile color, log2 (FPKM). (B) Heatmaps (p-val < 0.05) and bar diagrams (p-val < 0.05, FPKM > 0.5) shows the key genes screened from the leading-edge subset of ECM-Receptor Interaction and Focal Adhesion from GSEA. (C) Venn diagram shows the similarities and differences of key genes of ECM-Receptor Interaction and Focal Adhesion from 8 nm, 14 nm, and 20 nm vs. blank.

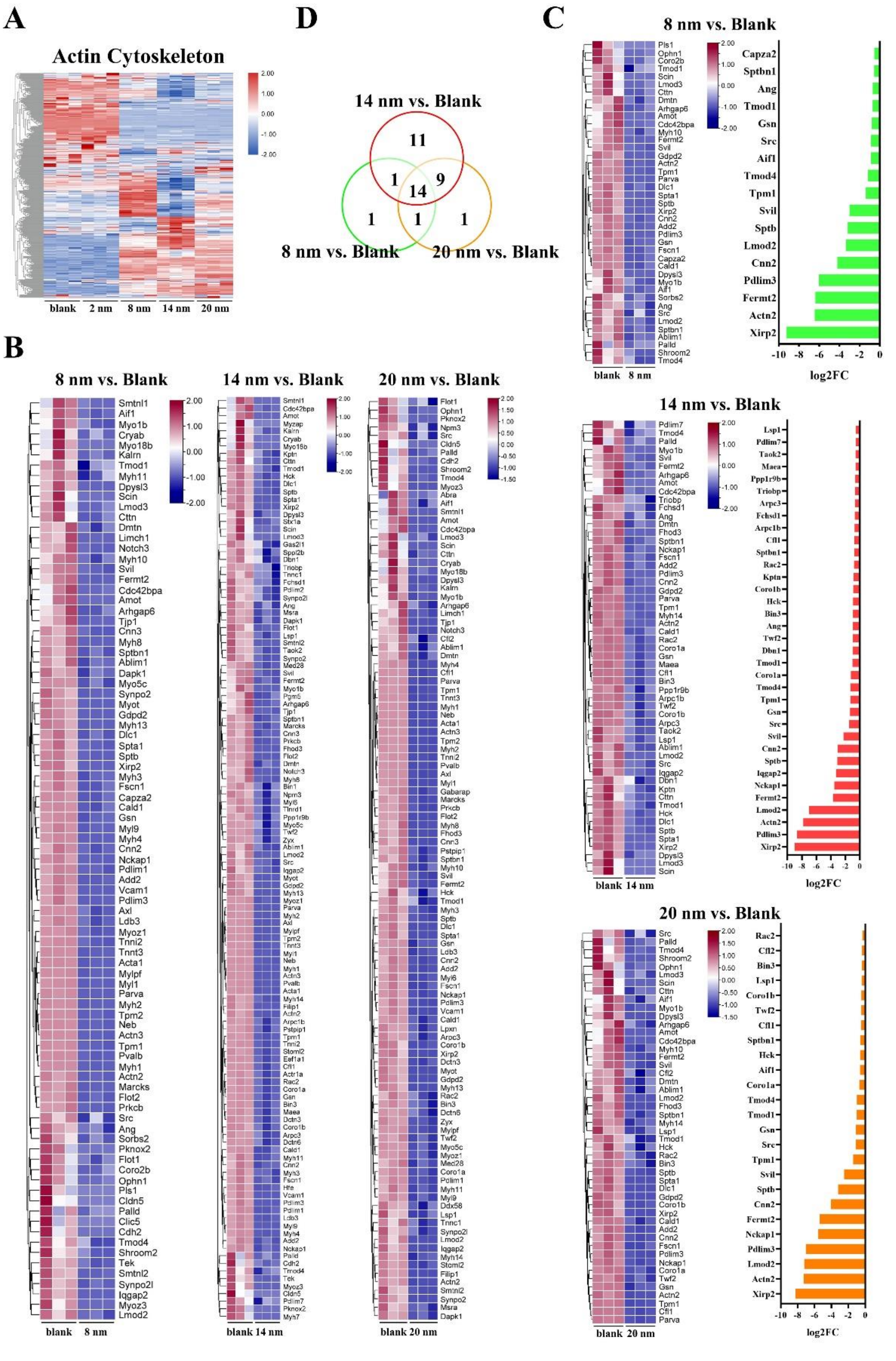

Figure S6. The expression differences of actin cytoskeleton related genes in 2 nm, 8 nm, 14 nm, 20 nm vs. blank. (A) Heatmap shows all genes expression from Actin Cytoskeleton. Tile color, log2 (FPKM). (B) Heatmaps (p-val < 0.05) show the key

genes screened from the leading-edge subset of Actin Cytoskeleton from GSEA. (C) Heatmaps (p-val < 0.05) and bar diagrams (p-val < 0.05, FPKM > 0.5) show the key genes involved in actin filament assembly related events from Figure 6C. (D) Venn diagram shows the similarities and differences of key genes of actin filament assembly from 8 nm, 14 nm, and 20 nm vs. blank.

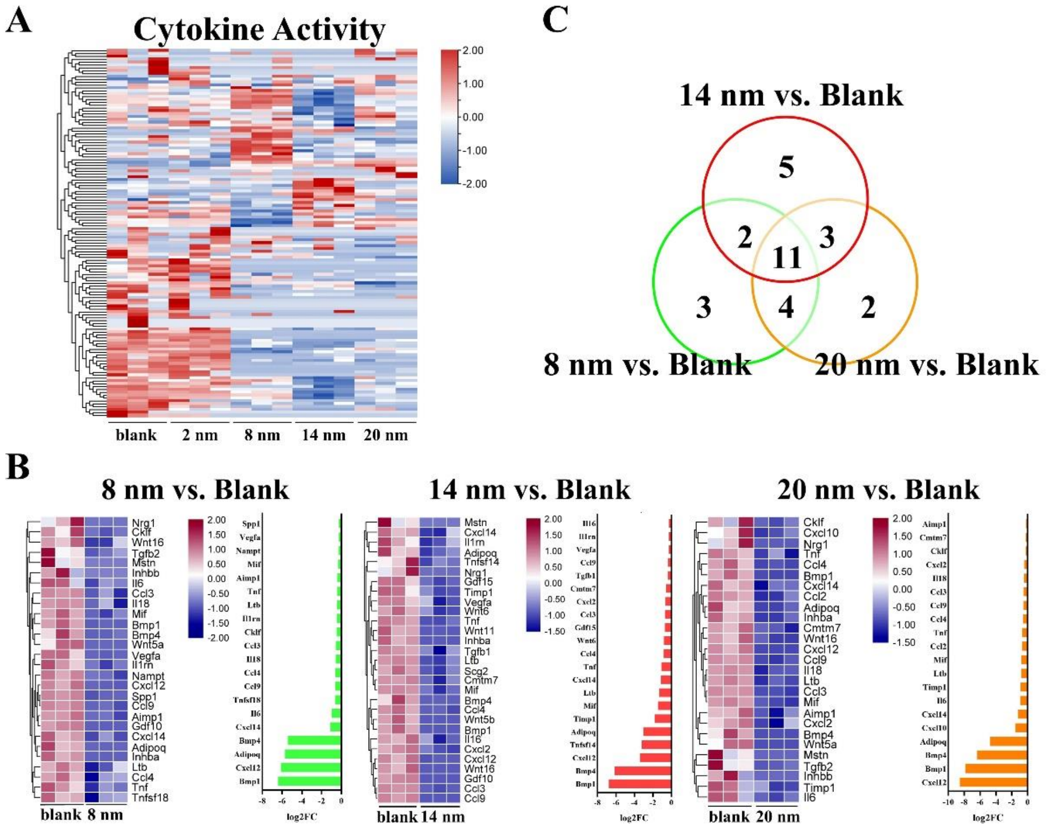

Figure S7. The expression differences of cytokine activity related genes in 2 nm, 8 nm, 14 nm, 20 nm vs. blank. (A) Heatmap shows all genes expression from Cytokine Activity. Tile color, log2 (FPKM). (B) Heatmaps (p-val < 0.05) and bar diagrams (p-val < 0.05, FPKM > 0.5) show the key genes screened from the leading-edge subset of cytokine activity from GSEA. (C) Venn diagram shows the similarities and differences of key genes of Cytokine Activity from 8 nm, 14 nm, and 20 nm versus blank.

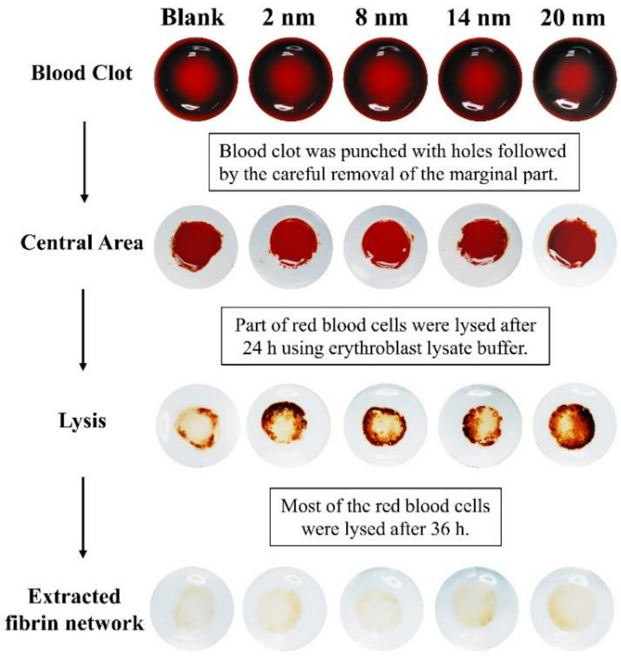

Figure S8. Fibrin network extraction procedures

Table S1. Silicon ion concentration in mesoporous silica/blood clot extracts. Data are presented as means ± s.d.; *n* = 3. One-way ANOVA with Tukey's *post hoc* test is applied.

Group	Si concentration (mg L^{-1})
Blank	/
2 nm	2.60±0.35
8 nm	5.84±1.49
14 nm	4.44±1.27
20 nm	4.63±1.57

Table S2. Primer pairs used in the RT-qPCR

Genes	Primer sequences
GAPDH-F	TCAGCAATGCCTCCTGCAC
GAPDH-R	TCTGGGTGGCAGTGATGGC
ITGB3-F	TAGGACTGAGAAGCAGAGGTG
ITGB3-R	CAGGTTACATCGGGGTGAGC
ZYX-F	TCTGTGCTGCTAGGTTGTGG
ZYX-R	CCCTTGATGTTTTTCCCCGT
FLNC-F	GGGTAACGTGGCCAACAAAC
FLNC-R	TTGGGGTTACAGGCTTCTGC
CAV1-F	GCAGCGCTGGAGTTTTCTGT
CAV1-R	GCCTGGCGCTACAACTTTTG
ITGB2-F	CTTCCTGGGATCTGAACTTCACT
ITGB2-R	TATCATCGGCTGGACAACCC
ITGAM-F	GAGGCCCCCAGGACTTTAAC
ITGAM-R	CTTCTTGGTGAGCGGGTTCT
ITGAV-F	CCAAAGGTGGCTCATGGGAT
ITGAV-R	CCACCACTTAAGACGGTCCC
VCL-F	TGGTCTAGCAAGGGCAATGA
VCL-R	CTCGTCACCTCATCAGAGGC
SRC-F	GCTCTTCGGAGGCTTCAACT
SRC-R	CTGACATCCACCTTCCTCGT
RAC2-F	GACAGTAAGCCGGTGAACCT
RAC2-R	TCCTTGTCATCGCGAAGGTC
HCK-F	GCTCCTTCATGATCCGGGAC
HCK-R	TGCCCAGCTCCAAGTTTCTT
ACTN2-F	CAACACTCCCAAACCCGATGA
ACTN2-R	GGGCTTATGCTTACGACGGT
Arpc3-F	AGTGCAACTCCAAGAGCCAA
Arpc3-R	CTCTCCGGGGATAGGGAAGT
CFL1-F	AGACAAGGACTGCCGCTATG
CFL1-R	GGCCCAGAAGATGAACACCA
TNF-F	CTGAACTTCGGGGTGATCGG
TNF-R	GGCTTGTCACTCGAATTTTGAGA
IL6-F	ATAGTCCTTCCTACCCCAATTTCC
IL6-R	GATGAATTGGATGGTCTTGGTCC
IL1B-F	TGGAGAGTGTGGATCCCAAG
IL1B-R	GGTGCTGATGTACCAGTTGG
MIF-F	TTCCACCTTCGCTTGAGTCC
MIF-R	GCATCGCTACCGGTGGATAA
CSF2-F	GGCTCACTGGCCCCATGTAT
CSF2-R	GAGTACTGGGCTCACTGCAA
TIMP1-F	CACACCAGAGCAGATACCAT

TIMP1-R	CCCTTATGACCAGGTCCGAG
SDF1-F	GACAAGTGTGCATTGACCCG
SDF1-R	CCTTGCATCTCCCACGGATG
CCL3-F	CAGCGAGTACCAGTCCCTTT
CCL3-R	GCAGTGGTGGAGACCTTCAT
CCL4-F	CCCAGCTCTGTGCAAACCTA
CCL4-R	GAGCAAGGACGCTTCTCAGT
CCL2-F	AGGTGTCCCAAAGAAGCTGT
CCL2-R	AAGACCTTAGGGCAGATGCAG
CCL9-F	TCGGTTTCCCAGCGGATTTT
CCL9-R	TGGCTTACTGATGGAGGGGT
